# Relationships Between Muscle Architecture, Deadlift Performance, and Maximal Isometric Force Produced at the Midthigh and Midshin Pull in Resistance-Trained Individuals

**DOI:** 10.1519/JSC.0000000000003455

**Published:** 2019-12-27

**Authors:** Sandro Bartolomei, Cosimo Rovai, Ivan Malagoli Lanzoni, Rocco di Michele

**Affiliations:** Department of Biomedical and Neuromotor Sciences, University of Bologna, Bologna, Italy

**Keywords:** force-time curve, maximal strength, muscle morphology

## Abstract

Bartolomei, S, Rovai, C, Lanzoni, IM, and di Michele, R. Relationships between muscle architecture, deadlift performance, and maximal isometric force produced at the midthigh and midshin pull in resistance-trained individuals. *J Strength Cond Res* 36(2): 299–303, 2022—The aim of this study was to investigate the relationships between muscle architecture, lower-body power, and maximal isometric force produced at midthigh pull (MTP), and at midshin pull (MSP). Twenty experienced resistance-trained men (age = 25.5 ± 3.2 years; body mass = 86.9 ± 12.4 kg; body height = 178.0 ± 5.3 cm) were tested for deadlift 1 repetition maximum (1RM), countermovement jump (CMJ), peak force (PF), and rate of force development (pRFD20) produced at isometric MTP and isometric MSP. Subjects were also assessed for architecture of vastus lateralis (VL). Physiological muscle thickness, pennation angle, and fascicle length (FL) were measured. Pearson's correlation coefficients were calculated to assess the relationships between variables. In addition, differences between MTP and MSP were assessed using paired-sample *t*-tests. A significant (*p* < 0.05) difference was detected on the correlation between deadlift 1RM and MSP (*r* = 0.78; *p* < 0.001) compared with MTP (*r* = 0.55; *p* = 0.012). Moderate correlations were observed between MSP PF and VLFL (*r* = 0.55; *p* = 0.011). Midshin pull pRFD20 was the only parameter significantly correlated with CMJ (*r* = 0.50; *p* = 0.048). Significantly higher PF and pRFD20 were recorded in MTP compared with MSP (*p* = 0.007 and *p* = 0.003, respectively). The present results show that force produced from the floor position may be more important than force produced from a position that mimics the second pull of the clean for deadlift and vertical jump performances. Coaches and scientific investigators should consider using MSP to assess isometric PF using a test correlated with both muscle architecture and dynamic performances.

## Introduction

Midthigh pull (MTP) represents a closed-chain isometric assessment of the ability of the neuromuscular system to apply force (18,23). The collection of the ground reaction force allows measurement of maximal isometric peak force (PF) and calculation of the rate of force development (RFD). Large correlations have been observed between isometric force produced at MTP and dynamic actions such as weightlifting (19,28) and throwing (37). Midthigh pull assessment is usually performed using knee-joint angles ranging from 120 to 145 (5,24). Comfort et al. (14) found no significant differences in PF and RFD from 120 to 150° of knee-joint flexion. Conversely, Marcora and Miller (26) reported significant changes in PF and RFD at isometric leg press using hip angles of 124 and 145°. To the best of our knowledge, only one study to date (7) performed the isometric pull from the regular barbell height at the beginning of the deadlift exercise (midshin pull [MSP]), characterized by a distance of 22.5 cm from the floor to the center of the bar. The authors reported significantly lower levels of PF produced from the floor position (MSP) compared with the regular MTP (7). Force produced at the beginning of the lift, however, may represent a key factor for success in deadlift, a discipline included in the international powerlifting program (2).

Ultrasonography has been widely used to assess muscle morphology in athletes and to evaluate morphological changes following resistance training (6). Although muscle cross-sectional area represents an important parameter for force production (34), recently, other parameters of muscle architecture such as pennation angle (PA) and fascicle length (FL) have been related to dynamic performances and agility (33,36,38). The aforementioned variables of muscle morphology can only be measured in pennated muscles (17). Vastus lateralis (VL) has been widely investigated in scientific studies using ultrasonography images. Significant correlations were detected between morphological characteristics of VL and maximal dynamic and isometric forces (3). Muscle architecture also demonstrated plasticity in response to resistance training programs (31). In particular, muscle thickness (MT) may be increased by resistance training, while changes in PA and FL may be more susceptible to high velocity and high rate of force production training (11,31).

The aim of the present investigation was to assess the relationships between MSP and MTP and the deadlift 1 repetition maximum (1RM). The similarity between MSP and the starting position of the deadlift exercise may suggest that larger correlations would be found between deadlift 1RM and MSP compared with MTP. Despite significant correlations have been detected between maximal isometric force produced at MTP and morphological characteristics of the VL (25,36,39), limited information exists about the relationship between muscle architecture and force produced in MSP. Thus, another aim of this study was to assess the relationships between muscle architecture and maximal isometric force produced at MSP and at MTP in resistance-trained individuals.

## Methods

### Experimental Approach to the Problem

Subjects reported to the laboratory on 2 separate occasions, 48 hours apart. In the first visit, they were assessed for muscle architecture VL and for maximal isometric strength. Maximal isometric strength tests, performed in randomized order, were MTP and MSP. In the second visit, subjects were asked to perform a countermovement jump (CMJ) test and the deadlift 1RM.

### Subjects

Twenty experienced resistance-trained men (mean ± *SD*: age = 25.5 ± 3.2 years; body mass = 86.9 ± 12.4 kg; body height = 178.0 ± 5.3 cm; body fat = 9.12 ± 4.13%; deadlift 1RM = 180.7 ± 27.1 kg) participated in this study. Subjects were resistance trained at least 3 times per week for more than 3 years (mean = 6.6 ± 3.5 years of experience) and were familiar with both powerlifting and weightlifting exercises. Inclusion criteria required subjects to be able to lift at least 2 times their body mass in deadlift. All the subjects signed an informed consent document after being informed about the risks and benefits of the study. Exclusion criteria included injuries occurred in the year before the study and the use of banned substances. Screening for performance enhancing drug use was accomplished using a health questionnaire completed at recruitment stage. Subjects were asked to abstain from alcohol, caffeine, and resistance training for at least 24 hours before the tests. The study was approved by the University of Bologna Bioethics Committee.

### Procedures

#### Strength and Power Testing

Anthropometric evaluations were performed before the first assessment session. Body measurements included body mass, height, and body fat composition. Body mass was measured to the nearest 0.1 kg using a scale (Seca 769; Seca Scale Corp., Munich, Germany). Body fat percentage was estimated from skinfold caliper measures using the method of Evans et al. (16). The same investigators performed all the skinfold analysis assessments. Before the strength and power assessments, subjects performed a standardized warm-up consisting of 5 min on a cycle ergometer against a light resistance, 10 body weight squats, 10 body weight walking lunges, 10 dynamic walking hamstring stretches, 10 dynamic walking quadriceps stretches, and 5 pushups (4).

Isometric maximal strength assessments consisted of an isometric MTP and an isometric MSP test performed in randomized order on a power rack that permitted fixation of the bar at a height that corresponded to the subject's midthigh and midshin, respectively, while standing on a force plate (Kistler, Kistler Force Plate; Winterthur, Switzerland, 500 Hz). For MTP, subjects were instructed to assume a body position similar to the second pull of the snatch and clean (140 and 125° angles for knees and hips, respectively). For both MTP and MSP, knee angle, hip angle, and grip width were measured using a goniometer and an anthropometric tape, respectively, to reproduce the same position for all testing sessions. For MSP, bar was set at a distance of 22.5 cm from the floor to the center of the bar to reproduce the official bar height in weightlifting and powerlifting competitions. During both MTP and MSP, subjects were secured to the bar using lifting straps and subsequently performed 3 maximal isometric pulls lasting for 6 seconds with a recovery time of 3 minutes between attempts (5). Tests were explained to the subjects before the beginning of the assessment session, and following the warm-up, each subject was asked to perform a familiarization trial including a submaximal isometric pull lasting for 6 seconds in both MTP and MSP. Subjects were required to assume a proper conventional deadlift technique, and sumo style was not allowed. For MTP and MSP, PF was recorded and RFD was calculated as previously described by Haff et al. (20). Peak RFD was calculated using a 20-ms window (pRFD20).

Intraclass coefficients were 0.94 (*SEM* = 158.4 N) and 0.99 (*SEM* = 32.56) for PF at MTP and MSP, respectively. Intraclass coefficients were 0.72 (*SEM* = 1,102.51 N·s^−1^) and 0.78 (SEM = 1,021.32) for pRFD20 at MTP and MSP, respectively. In addition, subjects were tested for CMJ using a contact mat (Globus Ergo Jump; Globus Ent, Codognè, Italy).

Deadlift 1RM test was performed as previously described by Hoffman (22). Each subject was asked to perform 2 warm-up sets using 40–60 and 60–80% of the perceived 1RM, respectively. Then, 3–4 subsequent trials were performed to determine the 1RM. The resting period between trials was set at 3–5 minutes.

#### Ultrasonography Measurements

Noninvasive skeletal muscle ultrasound images were collected from the subject's right thigh. Before image collection, all anatomical locations of interest were identified using standardized landmarks for the VL. The landmark for the VL was identified along its longitudinal distance at 50% from the proximal insertion of the muscle. The length of the VL encompassed the distance from the lateral condyle of the tibia to the most prominent point of the greater trochanter of the femur. Vastus lateralis measurement required the subject to lie on their side on the examination table for a minimum of 15 minutes before images were collected. The same investigator performed all landmark measurements for each subject.

A 12-MHz linear probe scanning head (Echo Wave 2; Telemed Ultrasound Medical System, Milan, Italy) was coated with water-soluble transmission gel to optimize spatial resolution and used to collect all ultrasound images. The probe was positioned on the surface of the skin without depressing the dermal layer, and the view mode (gain = 50 dB; image depth = 5 cm) was used to take panoramic pictures of the VL. During the measurements, subjects were asked to relax their leg muscles and maintain the left lateral decubitus position. Legs were positioned together, with a 10° bend angle in the knees (10). All images were collected and transferred to a personal computer. All ultrasound images were taken and analyzed by the same technician. Muscle thickness and PA were quantified in still images using the measuring features of the ultrasound device. Muscle thickness was determined as the distance between subcutaneous adipose tissue-muscle interface and intermuscular interface, and PA was determined as the angles between the echoes of the deep aponeurosis of the muscle and the echoes from interspaces among the fascicles. Fascicle length was calculated from MT and PA using the following equation (9):

$$\text{VLFL}=\text{MT}\times \text{sin}{\left(\text{PA}\right)}^{-1}.$$

Physiological muscle thickness (PMT) was calculated using the following equation (31):

$$\text{VLPMT}={\left({\text{MT}}^{2}+{\left[\text{tan}\;\text{PA}\times \text{MT}\right]}^{2}\right)}^{0.5}.$$

Intraclass correlation coefficients were 0.96 (*SEM* = 0.63 mm), 0.93 (*SEM* = 1.1°), and 0.96 (*SEM* = 8.0 mm) for VLPMT, VLPA, and VLFL, respectively.

### Statistical Analyses

A Shapiro-Wilk test was used to assess the normal distribution of the data. Differences between MTP and MSP were tested using paired-sample *t*-tests. In addition, effect size (Cohen's *d*) was used to evaluate differences between MSP and MTP. Pearson's correlation coefficients were used to examine selected bivariate relationships. According to Mukkaka (30), correlation coefficients (*r*) of 0.3, 0.5, 0.7, and 0.9 were interpreted as low, moderate, high, and very high relationship, respectively. Differences between the correlations of MSP PF and MTP PF with deadlift 1RM were statistically tested by means of the Steiger's Z test. All data are reported as mean ± *SD*. Significance was accepted at an alpha level of *p* ≤ 0.05.

## Results

All the data relative to performance assessments and muscle morphology were normally distributed (*p* = 0.05). Results for performance parameters and joint angles measured at MTP and MSP are reported in Table 1. Significant differences were detected between MSP and MTP for PF and pRFD20 (*p* = 0.007, *d* = 1.632 and *p* = 0.003, *d* = 0.965, respectively). Correlations among the principal parameters of performance and muscle architecture are reported in Table 2.

**Table 1:** Peak force (PF), peak rate of force development (pRFD20), and joint angles at midthigh pull (MTP) and at midshin pull (MSP) expressed as mean ± *SD*.

Isometric assessment	PF (N)	pRFD20 (N·s^−1^)	Knee angle (°)	Hip angle (°)
MTP	2,725.3 ± 536.6*	12,873.7 ± 4,959.3*	140	125
MSP	1967.2 ± 293.3	8,825.5 ± 3,247.0	73.2 ± 6.8	59.8 ± 4.8

* Significant difference (*p* ≤ 0.05) with MSP.

**Table 2:** Correlations among the principal parameters of performance and muscle architecture.*

Assessment	CMJ	MTP PF	MSP PF	Deadlift 1RM	VLPMT	VLPA	VLFL
CMJ							
*r*	1	0.09	−0.25	−0.21	−0.18	0.099	−0.38
*p*		0.707	0.284	0.380	0.432	0.679	0.098
MTP PF							
*r*		1	0.56	0.55	0.30	−0.17	0.368
*p*			0.011	0.012	0.196	0.473	0.111
MSP PF							
*r*			1	0.78	0.37	−0.33	0.55
*p*				0.000	0.107	0.161	0.011
Deadlift 1RM							
*r*				1	0.52	−0.09	0.57
*p*					0.020	0.682	0.008
VLPMT							
*r*					1	0.33	0.44
*p*						0.152	0.05
VLPA							
*r*						1	−0.51
*p*							0.022
VLFL							
*r*							1
*p*							

* CMJ = countermovement jump; MTP PF = midthigh pull peak force; MSP PF = midshin pull peak force; 1RM = 1 repetition maximum; VLPMT = physiological muscle thickness of vastus lateralis; VLPA = pennation angle of vastus lateralis; VLFL = fascicle length of vastus lateralis.

**Figure 1.**
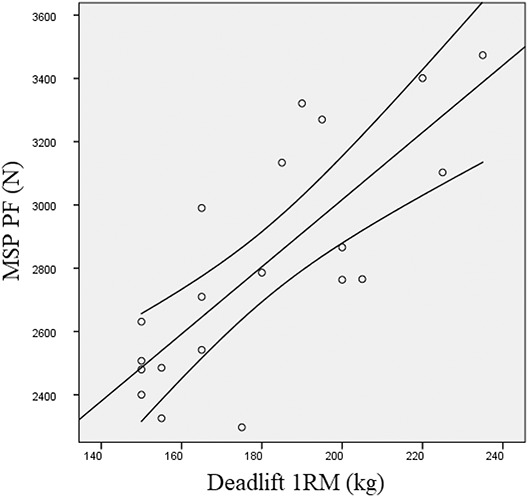
Scatter plots between MSP PF and deadlift 1RM. MSP PF = midshin pull peak force; 1RM = 1 repetition maximum.

**Figure 2.**
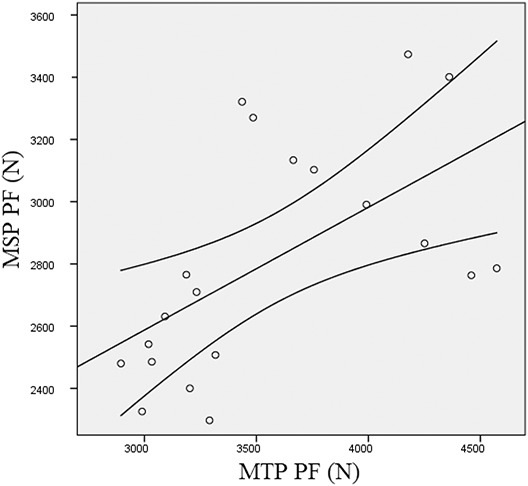
Scatter plots between MSP PF and MTP PF. MSP PF = midshin pull peak force; MTP PF = midthigh pull peak force.

Moderate correlations were observed between PF produced at MSP and MTP (*r* = 0.56; *p* = 0.011). Significantly higher correlations (*p* ≤ 0.05) were detected between deadlift 1RM and MSP PF (*r* = 0.78; *p* < 0.001) than between deadlift 1RM and MTP PF (*r* = 0.55; *p* = 0.012). Moderate correlations were also detected between MTP pRFD20 and MTP PF (*r* = 0.55; *p* = 0.015). Midshin pull pRFD20 was the only parameter significantly related with CMJ (*r* = 0.50; *p* = 0.048). No other significant correlations between strength parameters were noted. The scatter plots between MSP PF and deadlift 1RM and between MSP PF and MTP PF are shown in Figure 1 and Figure 2, respectively.

**Figure 3.**
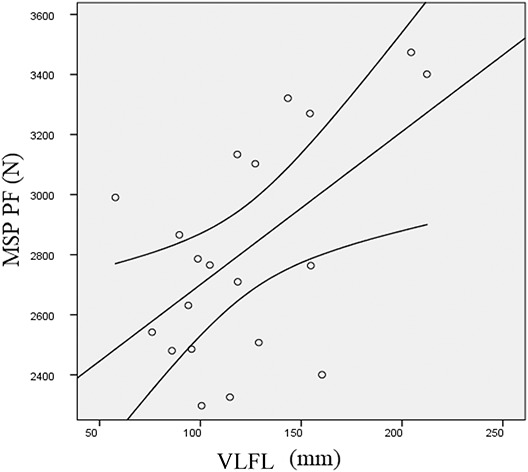
Scatter plots between MSP PF and VLFL. MSP PF = midshin pull peak force; VLFL = fascicle length of vastus lateralis.

All data of muscle architecture are displayed in Table 3. Moderate and low correlations were detected between VLFL and MSP PF (*r* = 0.55; *p* = 0.011) and between VLFL and MTP PF (*r* = 0.368; *p* = 0.111), respectively. Scatter plots between MSP PF and VLFL are shown in Figure 3. In addition, VLFL was also significantly correlated with deadlift 1RM (*r* = 0.57; *p* = 0.008). Moderate correlations were observed between VLPMT and deadlift 1RM (*r* = 0.52; *p* = 0.020). No other significant correlations were observed between performance parameters and muscle architecture.

**Table 3:** Parameters of muscle architecture measured on vastus lateralis (VL) expressed as mean ± *SD*.*

Parameter of muscle architecture of VL	
PMT (mm)	20.3 ± 3.3
PA ( ° )	9.9 ± 2.5
FL (mm)	122.0 ± 40.1

* PMT = physiological muscle thickness; PA = pennation angle; FL = fascicle length.

## Discussion

The results of this study showed that lower levels of isometric PF were produced in MSP compared with MTP by experienced resistance-trained individuals. This is consistent with Beckam et al. (7) who reported greater forces produced at MTP compared with lockout position (with hips almost extended) and with MSP. In MTP, subjects were able to use quadriceps and gluteus muscles to a greater extent compared with the floor position that characterizes MSP. Hip angle impacts the force produced by the lower body and in particular by the biarticular rectus femoris (13,15). In addition, different activations of both agonist and antagonist muscles were detected in deadlift at different hip angles (35). As reported by Hales et al. (21), the sticking region for the deadlift occurs approximately in correspondence to the knee level. During the MSP, the barbell is positioned below the knees while during the MTP, the barbell is above the knees. Both isometric assessments, therefore, are not performed in correspondence of the sticking point. In the present investigation, however, isometric PF produced at MSP, has been shown to be more related to deadlift 1RM compared with isometric PF expressed at MTP. Midshin pull represents the initial condition in which deadlift is performed during the 1RM or in competition. Muscle contractions before the movement of the bar indeed, are typically isometric. On the contrary, in the final phase of the deadlift, hip and knee extensions are mainly supported by concentric contractions (27). This difference in the contraction types between MTP and deadlift may have reduced the correlation between maximal strength produced in these exercises.

Previous investigations reported large correlations between MTP PF and weightlifting performance (8,19) such as snatch or clean and jerk. Maximal strength expressed in the second pull phase of the clean may be more important for final clean and snatch performances than the maximal force produced from the starting position. On the contrary, deadlift does not include the double-bend knee phase and the subsequent second pull phase (12) that characterizes the clean. This may explain the high importance of the force produced from the floor position and the high correlation detected between deadlift 1RM and MSP. Midthigh pull test may be more recommended for weightlifters interested in high pull forces produced above the knees. On the contrary, MSP may be more appropriate for athletes competing in powerlifting that requires high level of strength from the ground to accelerate the bar and to overcome the sticking point located in proximity to the knee level. Even if the pRFD20 values are spread out over a wide range in both MTP and MSP, significantly greater values were detected in MTP. Consistent with Nuzzo et al. (32), CMJ was not significantly related to maximal strength or to deadlift 1RM. In this study, MSP pRFD20 was the only parameter that was significantly related with CMJ. Interestingly, the same parameter calculated at MTP was not related with jumping performance. This is consistent with Kawamori et al. (24) who found no significant correlations between MTP pRFD20 and vertical jump performance in collegiate weightlifters. The lack of correlation may be due to the difference in hip and knee angles between the 2 exercises. On the contrary, MSP requires a deeper squatting position (73.2 and 59.8° for knee and hips, respectively) compared with MTP, more similar to that occurring in the CMJ. A limitation of the present investigation, however, is that joint angles in CMJ were not recorded.

The results of this study showed a moderate correlation between VLFL and PF produced at MSP. Correlation between VLFL and MTP PF however, were not significant. Longer FL may shift the force-length curve and influence the range of active force production (1). This may be an advantage when force is produced from a deep squatting position. Previous investigations reported significant correlations between VL FL and lower-body power (25,29), especially when resistance-trained individuals were tested. Despite the authors did not demonstrate whether higher FL were specific adaptations to resistance training or a genetic predisposition for power disciplines, Nimphius et al. (31) reported changes in muscle morphology in female athletes after 20 weeks of periodized training. Accordingly, Franchi et al. (17) reported significant increases in FL following 10 weeks of eccentric or concentric training. Interestingly, in the present investigation, PMT was significantly correlated with deadlift 1RM only. Despite muscle cross-sectional area represents the single most important determinant for muscle strength (34), other parameters of muscle architecture such as FL or PA seem to be related to dynamic or isometric performances when pennated muscles are taken into account. The large dispersion of scores within correlations between MSP PF and other parameters of maximal strength (Figures 1 and 2) and muscle architecture (Figure 3), may be due to anthropometric factors and technique. Even if subjects assumed a conventional deadlift position, maximum force expression may have been influenced by individual variations in pull technique.

**Practical Applications**

The use of the MSP may represent a valid alternative to the MTP to assess maximal isometric force capability in resistance-trained individuals. In particular, MSP seems appropriate to measure initial force in individuals competing in powerlifting events. The evaluation of muscle architecture may help to investigate individual muscle characteristics in relation to maximal strength and dynamic performances.
